# Supplementary material for: The relationship between alpha power and heart rate variability commonly seen in various mental states
Source: PLoS One. 2024 Mar 1;19(3):e0298961. doi: 10.1371/journal.pone.0298961 (PMC10906897; doi:10.1371/journal.pone.0298961)
Supplement: Attachment — Submitted filename: reply_plos_one.docx [file pone.0298961.s001.docx]

The followings are our point-to-point responses to the reviewers’ comments (the comments are shown with Italic and bold font).

Comments from Reviewer 1

***The introduction needs to be expanded with additional crucial information that is now missing. I suggest adding a section introducing and detailing what HRV is and its relevance within the context of this manuscript. It is currently only briefly mentioned, and this information is not sufficient to support the rest of the manuscript. Furthermore, it is recommended to search the literature for studies that investigated the relationship between HRV and alertness, just like the EEG evidence reported in the first paragraph.***

Thank you for your suggestion. We have followed your suggestion and added a definition of HRV. We have also added the following paragraph explaining that HRV reflects autonomic nervous system activity, citing the literature on the relationship between HRV and arousal level.

Heart Rate Variability (HRV) is the time variation between successive heartbeats, which is a beat-to-beat variation of R-R intervals. HRV analysis can assess the state of the autonomic nervous system [15]. The frequency feature in HRV is thought to be related to the dynamics of the sympathetic and parasympathetic nervous systems. While the low frequency (LF) band (0.04–0.15 Hz) reflects both sympathetic and vagal responses, the high frequency (HF) band (0.15–0.4 Hz) may be associated with parasympathetic control of the heart [15]. The relationship between HRV and emotional arousal has been studied. For example, Iwanaga et al. [16] showed that excited music increased the LF component of HRV, suggesting a decrease in parasympathetic nervous system activation. Another line of research reported that relaxing music increases parasympathetic nervous system activity, as reflected in HRV [17]. Thus, the quantification of HRV parameters can serve as an index of the autonomic nervous system’s status.

***The stimuli paragraph is a bit confusing. I suggest specifying that each of the eight pieces was created using a combination of six 30s samples. Moreover, the rationale for choosing these pieces should be further elaborated. Why specifically 8 of those were a combination of clips from the MER database? Were the remaining 4 pieces not given a valence-arousal value? If so, why not? Are these 4 pieces to be considered as control stimuli? Is there another study in the literature that has been used to guide the authors’ decisions? Authors write that the pieces from Naji’s work were chosen because they were less frequently heard, but based on which metrics? What is the benefit coming from the fact that these supposedly evoke no memories? I am sure the authors carefully considered these questions before implementing the study, but this information needs to be clearly spelled out for readers as well.***

Thank you for pointing this out. The explanation was inappropriate and has been corrected. The 12 experimental stimuli were selected aiming to cover all quadrants of emotional valence and arousal. First, for the eight experimental stimuli, six 30-second stimuli that were supposed to cover all quadrants were selected from the MER database to create a 3-minute video. Because the MER database contained a lot of music that was unfamiliar to the participants, such as ethnic music, we added 4 additional musical stimuli that we thought would be more familiar to the participants, based on Naji et al. The following modifications were made.

The experimental stimuli were selected with the aim of covering all quadrants of emotional valence and arousal. Twelve 3-minute music pieces (stimuli) that could evoke emotional responses were selected. For the eight music files, six 30s music clips were taken from the MER database [26] Because the MER database included a substantial amount of music that was unfamiliar to the participants, such as ethnic music, we supplemented it with four additional music stimuli that were familiar to them and likely to evoke emotion, based on Naji et al. [27].

***Page 6, music exposure paragraph: I believe there could be a more robust way of setting up the analysis, which could provide more interesting insights. If each track is assigned a valence-arousal value so that it falls in one of the four quadrants (but I am not sure of this, as the stimuli paragraph needs more details as mentioned above), then it would be possible to have four sets of tracks each composed of three tracks. This way, authors could run an ANOVA by considering the mean of each set instead of the twelve tracks individually. Moreover, this would make sense since tracks in each subset are expected to not be different among them. Finally, if the ANOVAs were to highlight significant differences, those should be further investigated with the use of post-hoc analyses.***

Thank you for pointing this out. The 12 stimuli were selected with the aim of covering all quadrants of emotional valence and arousal. As shown in the graph below, participants' ratings of arousal and valence for the 12 stimuli were not distributed in equal numbers in each quadrant separated by the means. Therefore, we did not perform the analysis you suggested, in which the stimuli were categorized by quadrant and averages were calculated.

Our goal was not to compare EEG and HRV between quadrants, but to distribute the arousal and valence ratings. Therefore, we report the results of the analysis as before. Note that although the main effect is significant, the post hoc analysis shows no significant difference between levels.

Fig. Scatter plot of the subjective value (arousal and valence) for each music stimuli. Dotted lines indicate the mean value.

***Page 6, line 32: authors state “as can be seen from the individual data points shown in the graphs, there appear to be individual variation”. I would argue that this is not a very sound methodology to discern eventual differences between conditions. I suggest implementing a more considerate approach when hypothesizing differences in the data.***

Thank you for your suggestion. To objectively show the variation of the data, we calculated the coefficient of variation (CV) for each indicator. Since there are several indicators for which the coefficient of variation is greater than 1.0, we can say that there is a large standard deviation from the mean. On this basis, we have changed the text as follows to claim that there are large individual differences in the change of the indicators.

As noted above, no significant differences were found in alpha power, HRV, or subjective reports when compared at rest before and after the task, suggesting that changes in these variables common across participants did not exist. Here, we calculated the coefficient of variation (CV) to assess the relationship between the index and their variability relative to the mean (Table 1). The coefficient of variation is the standard deviation divided by the mean. As can be seen from some CVs in these indices that are larger than 1.0, there appears to be individual variation in the pre- and post-rest changes in these indicators.

***The number of figures is overwhelming relative to the length of the manuscript. Some of these can be removed altogether, like figure 1, 3, and 4 since they report data with no significant differences. Figures 5, 6, and 7, provided the suggestion above relative to the analyses is implemented, could be merged into a single figure, as well as figure 10 and 11. This would bring the total number of figures to 5, which is more concise by delivering all the important information.***

Thank you for pointing this out. For data with no significant differences, we have removed the figures and presented the results in the text. Specifically, Figure 1 (subjective ratings of fatigue and sleepiness) and Figure 4 (HRV before and after the task), as well as Figure 5 (subjective ratings for each music group), Figure 6 (EEG for each music group), and Figure 7 (HRV for each music group) were removed and the mean and standard deviation information was added to the text.

Comments from Reviewer 2

***1. I assume the numbers in Figures 10 and 11 correspond to these results, but it's challenging to understand what was controlled for and how. It's crucial to clarify this, especially since the correlations prior to this did not yield a significant result.***

Thank you for pointing out that Figures 10 and 11 (Figures 5 and 6 in the revised manuscript) show the results of calculating partial correlations. In this partial correlation analysis, we computed partial correlations that control for the influence of other indicators when analyzing correlations between two indicators. The correlation analysis in Figures 8 and 9 (Figures 3 and 4 in the revised manuscript) is not a partial correlation, and the results differ from the partial correlation analysis because the influence of other indicators is not controlled.

***2. The term 'nHF' is not defined. How is it calculated? If it's a key result, its calculation method should be clearly explained.***

Thank you for pointing this out. The nHF calculation method was described in the Methods section, but it was written as "normalized LF" and did not include the abbreviation (i.e., nLF), which was confusing. We have corrected this as follows.

We calculated normalized LF and HF power, defined as the relative power of each component in proportion to total power minus VLF [32]: normalized LF (nLF) as the LF power in normalized units LF/(total power − VLF) × 100 and normalized HF (nHF) as the HF power in normalized units HF/(total power − VLF) × 100. The nLF and nHF mainly reflect sympathetic and parasympathetic responses, respectively. Normalization can eliminate much of the substantial variability in raw HRV spectral power, both within and across subjects [33].

***3. The rationale for choosing only male participants should be explained.***

Thank you for pointing this out. There was no reason to limit the participants in this study to males. However, since gender differences were reported, especially for HRV, we have added the following as a limitation of this study.

Finally, since all subjects are male, the extent to which our results can be generalized to women is limited. Recent meta-analysis suggests sex differences in HRV [66], therefore it is necessary to investigate the sex difference in heart-brain interaction observed in the current study.
