# Supplementary material for: The relationship between alpha power and heart rate variability commonly seen in various mental states
Source: PLoS One. 2024 Mar 1;19(3):e0298961. doi: 10.1371/journal.pone.0298961 (PMC10906897; doi:10.1371/journal.pone.0298961)
Supplement: Attachment — Submitted filename: reply_plos_one.docx [file pone.0298961.s002.docx]

The followings are our point-to-point responses to the comments (the comments are shown with Italic and bold font).

Comments from Reviewer 1

***- Results, HRV paragraph: Here you report that after the task nLF has (M = 60.9, SD***

***= 15.4, CV = 0.25), but this is a typo. The mean is 40.2 and the CV is 0.38 (taken***

***from the file uploaded to OSF). I suggest making a double check throughout the***

***manuscript to make sure all data has been reported correctly.***

Thank you for pointing this out. As you pointed out, the values in the text were incorrect and have been corrected. At the same time, the notation of the statistical tests was incorrect and has been corrected. We have rechecked all the results in the text to ensure that the rest is correct.

***- Figure 2 caption: change “Rectangles” to “Triangles”.***

Thank you for pointing this out. As you pointed out, the "rectangles" in the figure caption is incorrect and has been corrected to "triangles".
